# Analysis of ADAM12-Mediated Ephrin-A1 Cleavage and Its Biological Functions

**DOI:** 10.3390/ijms22052480

**Published:** 2021-03-01

**Authors:** Katsuaki Ieguchi, Takeshi Tomita, Toshifumi Takao, Tsutomu Omori, Taishi Mishima, Isao Shimizu, Massimiliano Tognolini, Alessio Lodola, Takuya Tsunoda, Shinichi Kobayashi, Satoshi Wada, Yoshiro Maru

**Affiliations:** 1 Department of Pharmacology, Tokyo Women’s Medical University, 8-1 Kawada-cho, Shinjuku, Tokyo 162-8666, Japan; tomori@twmu.ac.jp (T.O.); mishima130@gmail.com (T.M.); 2 Department of Clinical Diagnostic Oncology, Clinical Research Institute for Clinical Pharmacology and Therapeutics, Showa University, 6-11-11 Kita-karasuyama, Setagaya, Tokyo 157-8777, Japan; sst-wada@med.showa-u.ac.jp; 3 Institute for Biomedical Sciences, Interdisciplinary Cluster for Cutting Edge Research, Department of Biochemistry and Molecular Biology, School of Medicine, Shinshu University, 3-1-1 Asahi, Matsumoto, Nagano 390-8621, Japan; tomitat@shinshu-u.ac.jp; 4 Laboratory of Protein Profiling and Functional Proteomics, Institute for Protein Research, Osaka University, 3-2 Yamadaoka, Suita, Osaka 567-0871, Japan; tak@protein.osaka-u.ac.jp; 5 Department of Applied Chemistry, School of Advanced Science and Engineering, Waseda University, 3-4-1 Ohkubo, Shinjuku, Tokyo 169-8555, Japan; 6 Department of Food and Drug, University of Parma, Viale delle Scienze 27/a, 43124 Parma, Italy; massimiliano.tognolini@unipr.it (M.T.); alessio.lodola@unipr.it (A.L.); 7 Department of Medicine, Division of Medical Oncology, School of Medicine, Showa University 1-5-8, Hatanodai, Shinagawa, Tokyo 142-8555, Japan; ttsunoda@med.showa-u.ac.jp; 8 Clinical Research Institute for Clinical Pharmacology and Therapeutics, Showa University, 6-11-11 Kita-karasuyama, Setagaya, Tokyo 157-8777, Japan; s2koba@med.showa-u.ac.jp

**Keywords:** Eph, ephrin, ADAM, MMP, cancer, metastasis, biomarker, poor prognosis, urinalysis

## Abstract

Accumulating evidence indicates that an elevated ephrin-A1 expression is positively correlated with a worse prognosis in some cancers such as colon and liver cancer. The detailed mechanism of an elevated ephrin-A1 expression in a worse prognosis still remains to be fully elucidated. We previously reported that ADAM12-cleaved ephrin-A1 enhanced lung vascular permeability and thereby induced lung metastasis. However, it is still unclear whether or not cleaved forms of ephrin-A1 are derived from primary tumors and have biological activities. We identified the ADAM12-mediated cleavage site of ephrin-A1 by a Matrix-assisted laser desorption ionization mass spectrometry and checked levels of ephrin-A1 in the serum and the urine derived from the primary tumors by using a mouse model. We found elevated levels of tumor-derived ephrin-A1 in the serum and the urine in the tumor-bearing mice. Moreover, inhibition of ADAM-mediated cleavage of ephrin-A1 or antagonization of the EphA receptors resulted in a significant reduction of lung metastasis. The results suggest that tumor-derived ephrin-A1 is not only a potential biomarker to predict lung metastasis from the primary tumor highly expressing ephrin-A1 but also a therapeutic target of lung metastasis.

## 1. Introduction

A disintegrin and proteinases (ADAMs) represent a large family of enzymes (>50 in mammals), in which ADAM17, also known as TACE, shows protease activity against growth factors including HB-EGF and extracellular matrixes such as fibronectin. Approximately over half of all ADAMs lack proteinase activity and show proteinase-independent activity mediated through their disintegrin domains [1,2,3,4]. ADAMs play a role in rearrangement of the extracellular matrix and ectodomain shedding of growth factors, cytokines and GPI-anchored proteins [5]. Recent studies have found that ADAMs significantly contribute to tumor progression and metastasis and are up-regulated in various cancers such as breast cancer [6], glioblastoma multiforme (GBM) [7], and prostate cancer [8]. It has been reported that ADAM12 is up-regulated in bladder, gastric, lung, and breast cancer, and its high expression is associated with a poor prognosis [2]. Therefore, ADAM12 has been considered as a promising therapeutic target in cancer. However, no drugs targeting ADAMs are clinically used so far. Most of the molecular targeting drugs developed against MMPs and ADAMs show inhibitory effects with a broad-spectrum [9]. The presumed reason is supposed that substrate recognition sequences of ADAMs are not strongly restricted and relatively fuzzy compared with peptidases such as thrombin and Factor X. In fact, KB-R7785 was developed as a specific inhibitor for ADAM12 but it has shown widely inhibitory effects on ADAMs and MMPs activities.

Ephrin-A1 is a GPI-anchored protein localized in the external plasma membrane and it is a ligand for the type-A Eph receptor that is a transmembrane tyrosine kinase [10,11]. Moreover, ephrin-A1 was also identified as a soluble factor generated in response to TNFα stimulation [12]. EphAs and ephrin-As have been implicated not only in physiological events including embryonic and vascular development but also in some pathological features such as cancer and Alzheimer’s disease [13,14,15]. Analysis of clinical samples has demonstrated that up-regulation of ephrin-A1 is positively correlated with a poor prognosis in hepatocellular carcinoma and colon cancer [16,17]. We recently reported that ephrin-A1 up-regulated by S100A8—an endogenous ligand of the toll-like receptor 4 (TLR4)—is cleaved by ADAM12 in primary tumors. The ADAM12-mediated cleavage of ephrin-A1 induces vascular leakage in the lungs, resulting in an enhancement of tumor cell intravasation defined as metastasis [18,19]. It was also reported that soluble monomeric ephrin-A1 showed proliferative activity in some cultured tumor cells such as HeLa and SK-Br-3 cells [20]. Soluble forms of ephrin-A1 in serum were increased in tumor-bearing mice. Therefore, ephrin-A1 and the receptor, EphA2 are also promising targets for cancer therapy, and ephrin-A1 shows potential as a serum biomarker of cancer. We herein report a recognition system of ephrin-A1 by ADAM12 and confirmed that a soluble form of ephrin-A1 in serum and urine was derived from primary tumors. These results suggest tumor-derived ephrin-A1 would be a good candidate for a biomarker and therapeutic target against ephrin-A1-mediated metastasis.

## 2. Results

### 2.1. Identification of an ADAM12-Mediated Cleavage Site in Ephrin-A1 by Mass Spectrometry

**Figure 1:**
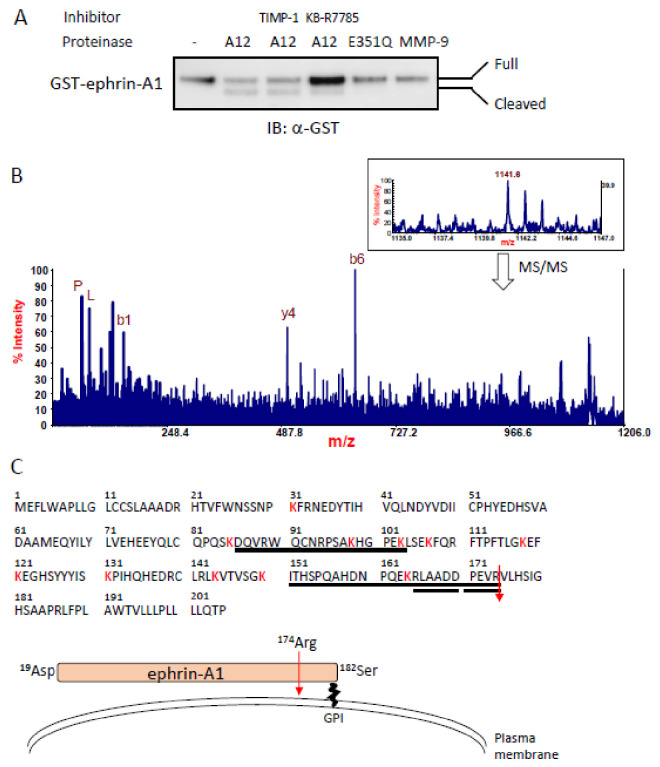
Mass spectrometric analysis of ADAM12-cleaved ephrin-A1. (**A**) ADAM12-mediated cleavage of ephrin-A1. GST-ephrin-A1 was incubated with recombinant human ADAM12-S. GST-ephrin-A1 was cleaved by ADAM12. KB-R7785 inhibited ADAM12-mediated ephrin-A1 cleavage but not TIMP-1, an MMP-9 inhibitor. (**B**) MALDI-MS (inset) and MS/MS spectra of the C-terminal LEP peptide of ephrin-A1. The protein was digested with LEP in buffer prepared with H_2_^18^O (see Materials and Methods). The digested peptides, but for the C-terminal one, were labeled at the C termini with ^18^O, which allowed for identification of the C terminal peptide of the protein as a non-labeled one. The ion peak at m/z 1141.6, which was observed as a non-labeled peak, could correspond to the peptide, R^165^LAADDPEVR^174^ (calcd. 1141.6, MH^+^). It was further confirmed by MS/MS, giving several sequence ions (b6: RLAADD, y4: PEVR, etc.) specific for the peptide [25]. Single characters (P, L) denote the immonium ions derived from the peptides. (**C**) Analysis of the C-terminal peptide of ephrin-A1 by MALDI-MS/MS. Cleaved ephrin-A1 was digested with LEP in buffer prepared with ^18^O-labeled water. The LEP peptide (R^165^LAADDPEVR^174^) was identified and observed as a non-labeled peptide by MALDI-MS/MS. The results indicate that ephrin-A1 was cleaved immediate after Arg at position 174 in ephrin-A1 amino acid sequence.

**Figure 2:**
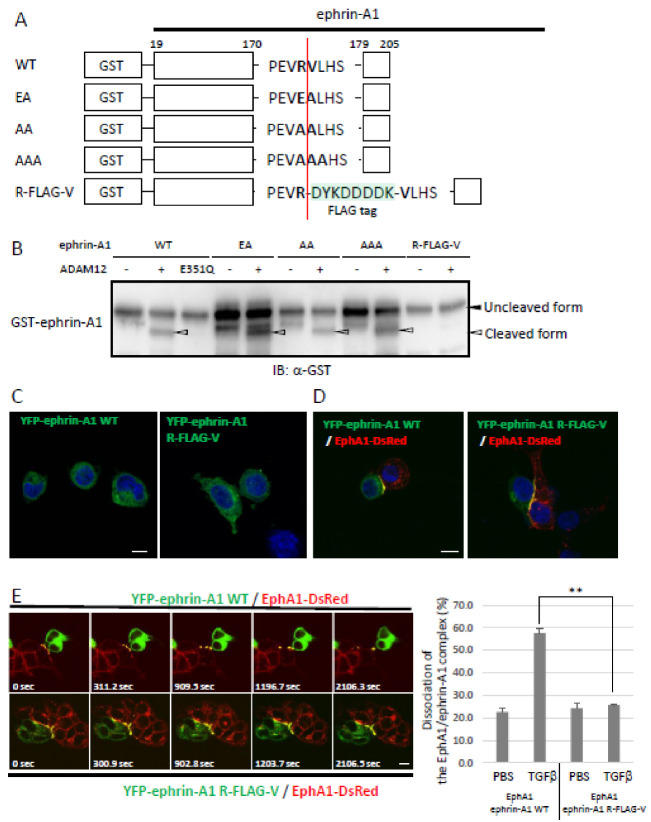
Mutational analysis of ADAM12-mediated ephrin-A1 cleavage. (**A**) A schematic presentation of ephrin-A1 mutants. (**B**) Effects of mutations in ephrin-A1 on ADAM12-mediated cleavage. Ephrin-A1 R-FLAG-V mutant was defective in ADAM12-mediated cleavage. (**C**) Subcellular localization of ephrin-A1 WT and R-FLAG-V mutant. HEK293 cells were transfected with YFP-ephrin-A1 WT or YFP-ephrin-A1 R-FLAG-V and observed by a confocal microscope. Both ephrin-A1 mutants showed similar subcellular localization in sparsely cultured conditions. (**D**) Binding capability of ephrin-A1 R-FLAG-V mutant against the EphA1 receptor. Co-localization of DsRed-tagged EphA1 and YFP-fused ephrin-A1 R-FLAG-V was found at cell boundaries and was equivalent to wild-type of ephrin-A1. Blue color shows DAPI signals. (**E**) Ephrin-A1 cleavage by ADAM12 in response to TGF-β stimulation and the quantification. Co-culture of EphA1-DsRed expressing cells and either YFP-ephrin-A1 WT or YFP-ephrin-A1 R-FLAG-V expressing cells was treated with 5 ng/mL TGF-β. Time-lapse images were obtained by a confocal microscope. Scale bars: 10 μm. Co-culture of EphA1-DsRed expressing cells and either YFP-ephrin-A1 WT or YFP-ephrin-A1 -R-FLAG-V expressing cells was treated with 5 ng/mL TGF-β. Dissociation of the EphA1/ephrin-A1 complex was counted under a fluorescence microscope. (*n* = 3, ** *p* < 0.01). Ephrin-A1 R-FLAG-V was defective in ADAM12-mediated cleavage induced by TGF-β.

We recently characterized ADAM12 as a protease toward GPI-anchored ephrin-A1 expressed in the plasma membrane [19]. In this study, we confirmed that a human-soluble form of ADAM12 lacking the transmembrane domain (ADAM12-S) but not ADAM12-S E351Q, a catalytically inactive mutant [21], cleaved GST-fused full-length human ephrin-A1 (GST-ephrin-A1) in vitro (Figure 1a). Debinski and his colleagues reported that commercially available recombinant MMP-9 also cleaved some ephrins including ephrin-A1 in vitro [22]. Therefore, we tested whether MMP-9 could cleave ephrin-A1. However, MMP-9 showed no effect on ephrin-A1 cleavage in our experimental conditions, while KB-R7785, a broad-spectrum ADAM inhibitor [23], significantly reduced ADAM12-mediated cleavage. The inhibitory activity was not observed using TIMP-1, an inhibitor of MMP-9 (Figure 1a). To identify the cleavage sites of ephrin-A1 recognized by ADAM12, GST-ephrin-A1 was incubated with ADAM12-S, and the cleavage products were digested by lysylend-peptidase (LEP). To distinguish the ADAM12 cleavage sites from those of LEP, the digestion was carried out in buffer prepared with 65 atom% H_2_^18^O. In this condition, all the LEP-digested peptides, except for the C-terminal one, partially incorporated ^18^O (~65%) atoms at the C-terminal carboxyl groups, which showed doublet isotopic peaks owing to the ^18^O labeling. On the other hand, the original C-terminal peptide, which should not incorporate ^18^O atom and give the natural isotope peak pattern, was observed at m/z 1141.6 (inset of Figure 1b). Its MS/MS spectrum clearly gave a signature of the peptide to be RLAADDPEVR^174^ (Figure 1b), implying that the ephrin-A1 was cleaved at R^174^ by ADAM12 (Figure 1b,c). We obtained the same results for ephrin-A1 purified from HEK293T cells stably expressing human ephrin-A1 (data not shown). These results suggest that ADAM12 specifically cleaves ephrin-A1 in our experimental conditions. We hereafter define ADAM12-cleaved ephrin-A1 as ephrin-A1 174R (Asp19-Arg174). Subsequently, we inserted mutations around the ADAM12-mediated cleavage site of ephrin-A1 (Figure 2a). In agreement with the results of the ADAM10-mediated cleavage of ephrin-A5 [24], the point mutations around the cleavage site had no effect on the cleavage of ephrin-A1 by ADAM12 (Figure 2b). ADAM10 lost its cleavage capacity toward ephrin-A5 by an insertion of FLAG tag sequences into the cleavage site for ADAM10. Similarly, we also inserted the FLAG tag to the cleavage site of ephrin-A1 recognized by ADAM12. The ephrin-A1 mutant (R-FLAG-V) abrogated ADAM12-mediated ephrin-A1 cleavage (Figure 2b) but other mutants did not. Ephrin-A1 R-FLAG-V showed no difference of subcellular localization and its binding ability to the EphA1 receptor (Figure 2c,d). Boundary formation by interactions between YFP-ephrin-A1 WT and EphA1-DsRed was dissociated after 2106 s upon TGF-β stimulation but Ephrin-A1 R-FLAG-V stayed together with EphA1-DsRed at the cell boundaries. (Figure 2e) [19]. Boundary formation by interactions between YFP-ephrin-A1 WT and EphA1-DsRed was dissociated 2106 s after TGF-β stimulation but Ephrin-A1 R-FLAG-V stayed together with EphA1-DsRed at the cell boundaries.

### 2.2. Bioactivity of ADAM12-Cleaved Ephrin-A1 on the Eph/Ephrin Signal

**Figure 3:**
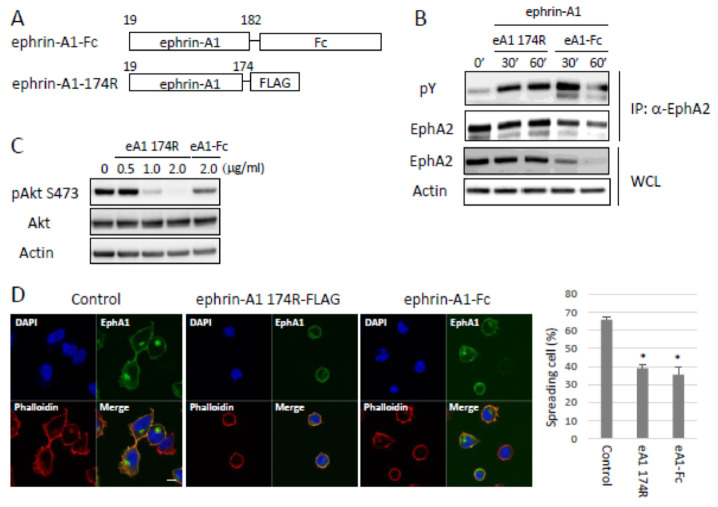
Bioactivity of ephrin-A1-174R. (**A**) A schematic presentation of soluble Figure 1. Fc-fused ephrin-A1 is commercially available. FLAG-tagged ephrin-A1 was produced in HEK293 cells and purified with FLAG antibody. (**B**) Tyrosine phosphorylation of EphA2. HEK293 cells expressing EphA1-EGFP were stimulated with ephrin-A1-174R for indicated time points. Ephrin-A1 174R induced tyrosine phosphorylation of EphAs, and the phosphorylation levels are comparable to those of ephrin-A1-Fc. (**C**) Dephospholylation of Akt upon ephrin-A1 stimulation. Both ephrin-A1 174R and Fc-fused ephrin-A1 induced dephosphorylation of Akt. (**D**) Cell-spreading assay on ephrin-A1-coated culture dish and the quantification. EphA1-EGFP expressing HEK293 cells were seeded onto either ephrin-A1 174R or ephrin-A1-Fc-coated well. Scale Bars: 10 μm. Evaluation of cell-spreading defects. More than 100 cells were counted and evaluated for whether there was spread or not. Ephrin-A1 174R induced cell-spreading defects as well as ephrin-A1-Fc. (* *p* < 0.05).

To test the biological activities of ephrin-A1 174R, we compared the activation capability on EphA2 tyrosine phosphorylation and EphA2 downstream signal between a commercially available recombinant Fc-fused ephrin-A1 Asp19-Ser182 (ephrin-A1-Fc) and ephrin-A1 174R (Figure 3a). We stimulated HEK293 cells with ephrin-A1-Fc or ephrin-A1 174R. Ephrin-A1 174R induced tyrosine phosphorylation of EphA2 at comparable levels to those by ephrin-A1-Fc (Figure 3b) and strongly dephosphorylated Akt but slightly dephosphorylated Akt by ephrin-A1-Fc (Figure 3c). Induced-serine dephospholylation of Akt by ephrin-A1 stimulation is consistent with a previous study [26]. We previously reported that EphA1-GFP expressing HEK293 cells were defective in cell-spreading on ephrin-A1-Fc-coated wells in a cell culture plate [27]. Therefore, we checked the effect of ephrin-A1 174R stimulation on cell-spreading ability. Ephrin-A1 174R showed cell-spreading defects as well as ephrin-A1-Fc (Figure 3d). Taken together, these results indicate that ephrin-A1 174R is biologically active at least against EphA1 and EphA2. We tried to perform in vitro assay using HEK293 cells, as shown in Figure 2e and Figure 3e, also in 3LL cells. However, 3LL cells did not allow us to carry out the experiments because they showed no response to TGFβ stimulation and cell-spreading defects without ephrin-A1 stimulation. At least in HEK293 cells, ephrin-A1 174R had biological activities, and TGFβ stimulation-induced ADAM12-mediated ephrin-A1 cleavage.

### 2.3. Effects of Ephrin-A1 Cleavage on Tumor Growth and Metastasis

**Figure 4:**
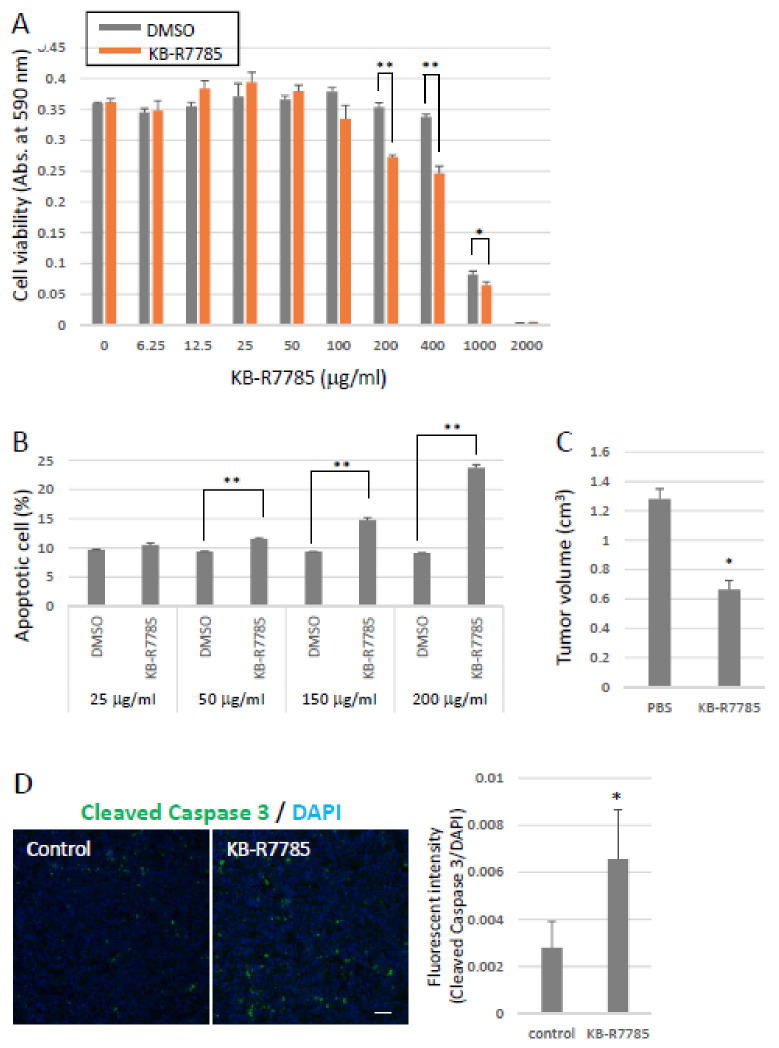

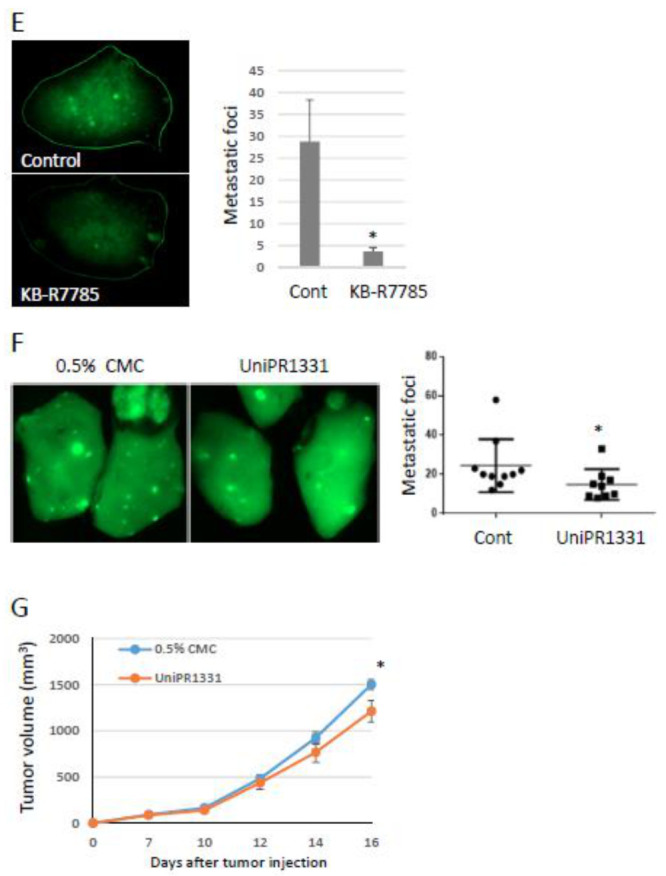
The effects of inhibitors against the ADAM12/ephrin-A1/EphAs axis on tumor growth and metastasis. (**A**) Effects of KB-R7785 on cell viability in vitro. MTT assay was performed. KB-R7785 significantly decreased cell viability. (* *p* < 0.05, ** *p* < 0.01) (**B**) Effects of KB-R7785 treatment on apoptosis in vitro. Apoptosis was checked by a flow cytometer using Annexin V. (**C**) Effects of KB-R7785 treatment on tumor growth. C57BL/6 WT mice were subcutaneously inoculated with LLC cells and intraperitoneally injected with KB-R7785. Tumor size was measured by a caliper. KB-R7785 treatment attenuated tumor growth. (* *p* < 0.05). (**D**) Immunostainings of the primary tumors using an anti-cleaved caspase-3 antibody and the quantification. Evaluation of cleaved caspase-3 in the primary tumors. Green signals (cleaved-caspase-3) were evaluated by ZEN image browser. At least five frames per tumor were evaluated. Apoptosis was obviously enhanced in KB-R7785-treated tumors. (*n* = 5, * *p* < 0.05, ** *p* < 0.01). Scale Bars: 10 μm (* *p* < 0.05, ** *p* < 0.01) (**E**) Effects of KB-R7785 treatment on spontaneous lung metastasis. C57BL/6 WT mice were subcutaneously inoculated with 3LL-EGFP cells and intraperitoneally injected with KB-R7785. Metastatic foci were observed and counted under a fluorescence stereoscopic microscope. KB-R7785 treatment significantly reduced metastatic foci. (*n* = 9–10, * *p* < 0.05) (**F**) The effect of pan EphA inhibitor on lung metastasis and (**G**) tumor growth. C57BL/6 mice were subcutaneously inoculated with 3LL-EGFP cells and administrated with UniPR1331 (3 mg/kg) every day for three weeks per os. Metastatic foci were observed and counted under a fluorescence stereoscopic microscope. Treatment with UniPR1331 significantly decreased the number of metastatic foci in the lungs and tumor growth. (*n* = 9–10, * *p* < 0.05).

To investigate the effect on the inhibition of ADAM12 in tumor growth, we performed MTT assay to check the anti-proliferative activity of KB-R7785 against 3LL, a highly metastatic Lewis lung carcinoma cell. KB-R7785 treatment significantly inhibited cell viability and enhanced apoptosis (Figure 4a,b). Subsequently, we tested the inhibitory effect of KB-R7785 on tumor growth in mice. C57BL/6 mice were subcutaneously inoculated with 3LL cells stably expressing EGFP and were subsequently intraperitoneally injected with KB-R7785 every other day for 16 days. KB-R7785 treatment markedly inhibited primary tumor growth (Figure 4c) and enhanced apoptosis in the primary tumors (Figure 4d). Moreover, we found a significant decrease of lung metastasis in KB-R7785-treated mice (Figure 4e). The anti-proliferative activity of KB-R7785 might be due to an inhibition of ephrin-A1 cleavage. We also checked the effect of soluble ephrin-A1-EphA receptor interactions on metastasis using UniPR1331, a cholenic acid-based EphA broad-spectrum antagonist [28]. Treatment of UniPR1331 successfully inhibited lung metastasis (Figure 4f) and slightly but significantly inhibited tumor growth (Figure 4g). These results indicate that an inhibition of ephrin-A1 function has a therapeutic potential for lung metastasis.

### 2.4. Tumor-Derived Ephrin-A1 as a Putative Biomarker for Metastasis

**Figure 5:**
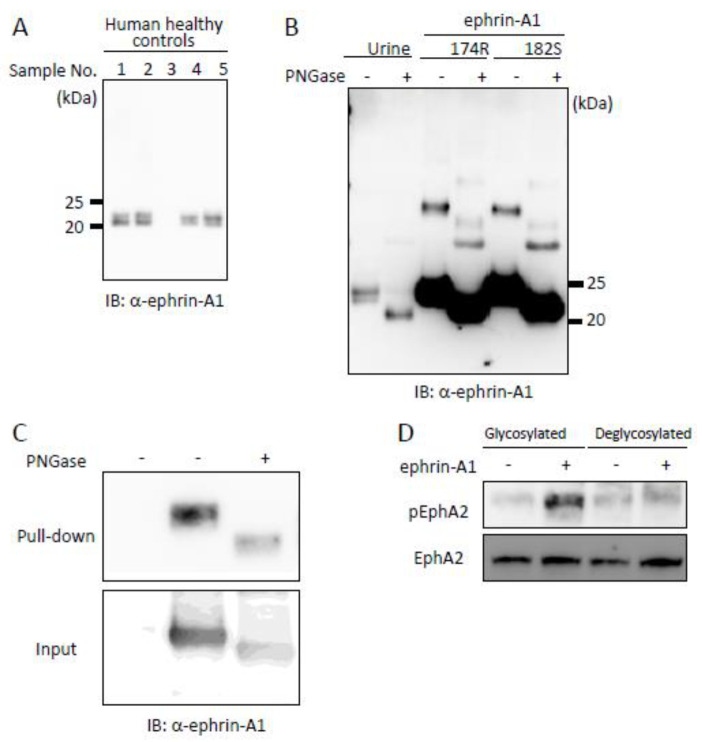
Effects of glycosylation of ephrin-A1 on the receptor activation. (**A**) Ephrin-A1 in urine of human healthy controls. Fifty-fold times concentrated human urine was analyzed by immunoblotting using anti-ephrin-A1 monoclonal antibody. Urinary ephrin-A1 was detected. (**B**) Comparison of recombinant human ephrin-A1 produced in HEK293 cells and human urinary ephrin-A1. Ephrin-A1 174R and urinary ephrin-A1 were treated with PNGase and analyzed by immnoblotting. Both were glycosylated due to post-translational modification (**C**) Effect of glycosylation on ephrin-A1 on the receptor binding. Either glycosylated ephrin-A1 or deglycosylated ephrin-A1 was incubated with His-tagged extracellular domain of EphA2. Pull down assay indicated that deglycosylation of ephrin-A1 showed no effect on its receptor binding. (**D**) Effect of glycosylation on the receptor activation. HEK293 cells were stimulated with either glycosylated or deglycosylated ephrin-A1. Deglycosylated ephrin-A1 failed to induce tyrosine phosphorylation of EphA2.

**Figure 6:**
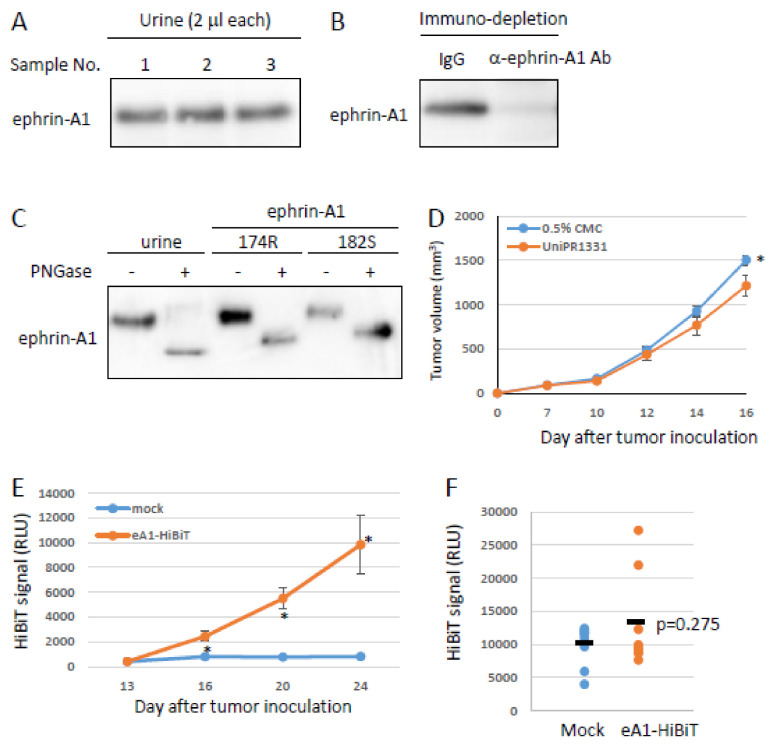
A putative biomarker of lung metastasis. (**A**) Urinary ephrin-A1 in mice. Urine was collected from healthy mice and analyzed by immunoblotting. Two microliters of urine were used for immunoblotting. (**B**) Immuno-depletion of urinary ephrin-A1. Two microliters of urine were incubated with anti-ephrin-A1 antibody. The immune complex was precipitated with Protein G beads, and the supernatant was analyzed by immunoblotting. Urinary ephrin-A1 was disappeared by an incubation with anti-ephrin-A1 antibody. (**C**) Glycosylation of mouse urinary ephrin-A1. Mouse urinary ephrin-A1 was treated with PNGase. Mouse urinary ephrin-A1 was shifted down. (**D**) Effects of soluble ephrin-A1 174R on tumor growth. LLC cells stably expressing a soluble form of HiBiT-tagged ephrin-A1 174R were subcutaneously inoculated into B6 mice. Tumor mass was measured by a caliper, and (**E**) serum and (**F**) urine were collected from the mice twice a week. Serum levels of HiBiT-ephrin-A1 were markedly elevated 16 days after tumor inoculation. HiBiT-ephrin-A1 in urine was detected in some mice but not all. (*n* = 8, * *p* < 0.05).

We previously reported that soluble forms of ephrin-A1 were increased in the serum of tumor-bearing mice [19]. The establishment of a detection system for soluble forms of ephrin-A1 would lead to develop a clinical test for the prediction of metastatic potency. To test this possibility, we tried to detect soluble forms of ephrin-A1 in urine because urinalysis is the easiest and cheapest biopsy. To confirm the existence of soluble forms of ephrin-A1 in urine samples, we performed immunoblotting by using the urine samples of human healthy controls. A soluble form of ephrin-A1 was found in urine (Figure 5a). Ephrin-A1 is thought to be glycosylated after its translation and an important event for the receptor activation. To examine if urinary ephrin-A1 has undergone the translational modification, urinary ephrin-A1 was treated with PNGase. Ephrin-A1 bands were shifted down by deglycosylation (Figure 5b). Subsequently, we tested the effect of the glycosylation on ephrin-A1 on the binding to the EphA2 receptor and the activation capability. Deglycosylated ephrin-A1 bound to the EphA2 receptor but failed to activate (Figure 5c–d). These results suggested that soluble ephrin-A1 in urine is biologically active. To examine the possibility of ephrin-A1 as a biomarker, we checked if ephrin-A1 is found in mouse urine. Ephrin-A1 was found in urine derived from healthy mouse as detected in human urine (Figure 6a). We also performed immuno-depletion assay and mass spectrometric analysis to confirm if the bands detected by immunoblotting in Figure 6a are ephrin-A1. The ephrin-A1 band disappeared by an incubation with anti-ephrin-A1 antibody (Figure 6b). Mouse urinary ephrin-A1 is glycosylated as observed in human urine (Figure 6c). Urinary ephrin-A1 purified by using Concanavalin A beads was analyzed by mass spectrometry. A Mascot database search, based on the mass spectra of the LEP digest of ephrin-A1, revealed a partial ephrin-A1 specific peptide (Supplementary Figure S1). Taken together, we demonstrated that ephrin-A1 exists in mouse urine. Subsequently, we checked if soluble forms of ephrin-A1 derived from the primary tumors (tumor-derived ephrin-A1) is released to urine. We subcutaneously injected LLC cells stably expressing HiBiT-tagged soluble ephrin-A1 (ephrin-A1-HiBiT) into mice and collected their urine. Ephrin-A1-HiBiT can exclude endogenous ephrin-A1 and be detected as a luminescence using a specific peptide that interacts with HiBiT tag [29]. Tumor-derived soluble ephrin-A1 levels were gradually increased in serum along with the growth of tumor mass (Figure 6d–e). Tumor-derived ephrin-A1 in urine was observed in some, but not all mice (Figure 6f). The data suggest that urinary ephrin-A1 levels would be a potential prognostic marker for metastasis.

## 3. Discussion

KB-R7785, a broad-spectrum inhibitor for ADAMs, significantly inhibited lung metastasis and simultaneously reduced tumor growth through an enhanced apoptosis. An inhibition of tumor growth by KB-R7785 treatment is unexpected for us, but it is consistent with previous reports published elsewhere [27,30]. Moreover, we previously reported that treatment with a neutralizing antibody against soluble forms of ephrin-A1 inhibited primary tumor growth [19], and another group reported that soluble ephrin-A1 induced cell proliferation in some human breast cancer cell lines [31]. As a considerable mechanism of the KB-R7785-mediated inhibition of cell proliferation, ADAMs regulate cleavages of growth factors such as HB-EGF and IGF. These growth factors are well-known as inducible factors of tumor growth. Therefore, KB-R7785 may inhibit Erk signaling induced by growth factors for cell proliferation [32]. Moreover, ADAMs show protease activity toward ECM such as fibronectin and collagen. These substances are required for the establishment of tumor development and the maintenance of cancer stem cell properties [33]. A reduced tumor growth by KB-R7785 treatment may be due to combined effects as described above. MMPs and ADAMs have been considered as undruggable targets for a long time, and there is no drug clinically used so far [9]. Moreover, they have no shared conserved sequences that recognize the substrate and not specifically work. The molecular targeting drugs could be developed like a decoy peptide drug when we find a specific peptide sequence recognized by each MMP or ADAM protease. 

Some ephrin-A1 cleavage sites recognized by MMP-1, MMP-2, MMP-9, and/or MMP-13 were already reported by mass spectrometric analysis using culture supernatant of U-251 MG cell exogenously expressing ephrin-A1 [22]. However, a specific cleavage site recognized by individual protease is still unsolved and no evidence of a specific cleavage site of ephrin-A1 by MMPs in U-251 MG cell exogenously expressing ephrin-A1 was provided. In this paper, we characterized for the first time the specific cleavage site of ephrin-A1 by an individual protease. In our in vitro experiments, MMP-9 somehow showed no protease activity toward ephrin-A1. The reaction buffer and recombinant protein of ephrin-A1 and MMP-9 using in vitro cleavage assay may cause the difference. We also herein demonstrate ephrin-A1 174R has a comparable biological activity to that of commercially available ephrin-A1-Fc. Although multimeric or dimeric ephrin-A1-Fc has been used for many Eph/ephrin studies, ephrin-A1 does not exist as an Fc-fusion protein in physiological and pathological conditions. In fact, only ephrin-A1 was abundantly detected by immunoblotting around the 25 kDa protein marker in the urine of healthy mice. Nevertheless, the source of urinary soluble ephrin-A1 is unknown. We suppose that the urinary ephrin-A1 is derived from kidney because immunofluorescence staining showed that ephrin-A1 is expressed in the tubular epithelium and glomerulus based on immunostainings in the human protein atlas database (https://www.proteinatlas.org/ENSG00000169242-EFNA1/tissue/kidney#img; accessed on 1 March 2021). The physiological functions of urinary ephrin-A1 are still unclear although urinary ephrin-A1 should have biological activities because it was glycosylated in humans and mice. We established that both tumor-derived soluble ephrin-A1 and tissue-derived soluble ephrin-A1 were gradually increased in serum concomitant with tumor growth. However, we could not detect tumor-derived ephrin-A1 in the urine of all tumor-bearing mice. The HiBiT signal in urine was approximately 10 times lower than that in PBS when HiBiT-tagged ephrin-A1 was mixed with mouse urine. We suppose that unknown factors in urine interrupt the substrate reaction for HiBiT luminescent signal. Therefore, we could not detect a HiBiT signal in all urine samples derived from tumor-bearing mice due to technical issues. Urinalysis is the easiest, less stressful type of biopsy. Tumor-derived ephrin-A1 is completely distinguishable from physiological soluble ephrin-A1 in experimental mouse models but not in pathological conditions in human. Furthermore, we cannot specifically detect ADAM12-mediated soluble ephrin-A1 in urine although some soluble forms of ephrin-A1 were confirmed in human healthy donors and the in vitro study reported by Beauchamp et al [22]. This is the biggest problem that needs to be solved in this study. Therefore, further investigations will be required to establish urinary ephrin-A1 as a biomarker for metastasis. It was reported that ephrin-A1 was found by a dot blot assay using serum derived from hepatocellular carcinoma patients [34]. Moreover, we also reported that the soluble form of ephrin-A1 was increased in the serum of tumor-bearing mice [19]. Serum ephrin-A1 would also be a good candidate for a biomarker. It has been well-known that serum protein is altered in glycan biosynthesis such as the enhancement of fucosylation in cancer patients [35,36]. This might be a good candidate to exclude the soluble ephrin-A1 in serum or urine in physiological conditions. Consequently, ELISA or immuno-chromatographic assay using ephrin-A1 antibody in combination with a specific antibody against ephrin-A1 glycans would be the best approach for a prediction of metastasis. Successful metastatic prediction will provide a good outcome and improve the prognosis for advanced cancer patients.

## 4. Materials and Methods 

### 4.1. Materials

Antibodies against FLAG were purchased from Fujifilm Wako (Osaka, Japan). Recombinant ephrin-A1-Fc and MMP-9 were purchased from R&D Systems (Minneapolis, MA, USA). A monoclonal antibody against mouse ephrin-A1 and a polyclonal antibody against human ephrin-A1 were purchased from Santa Cruz Biotechnology (SC-37732 and SC-911, Santa Cruz, CA, USA). Anti-EphA2, anti-phospho-EphA2 (Y588), anti-Actin, anti-Akt, and anti-phospho-Akt (S473) antibodies were purchased from Cell Signaling Technology (Danvers, MA, USA). 

### 4.2. Plasmids

DsRed-tagged and EGFP-tagged human EphA1-WT were subcloned into pDsRed-monomer N1 or pEGFP-N3 vectors (Takara Bio, Shiga, Japan) as previously described [19]. Human YFP-tagged ephrin-A1 was constructed as previously described [19]. N-terminal GST- and C-terminal FLAG-tagged ephrin-A1 (19–205aa and 19-174) were subcloned into pGEX6P-1 (GE Healthcare, Uppsala, Sweden) and pcDNA3.1 vector, respectively. Site-directed mutagenesis was performed using KOD-plus Mutagenesis kit to construct ephrin-A1 mutants and ADAM12-S E351Q inactive mutant (TOYOBO, Tokyo, Japan). Human ADAM12-S was kindly provided from Dr. Ulla Wewer at the University of Copenhagen.

### 4.3. Preparation of Recombinant Protein

Human ADAM12-S was transiently transfected into CHO cells by NEPA21 (Nepagene, Chiba, Japan). The cells were cultured in serum-free medium for 48 h. Subsequently, the supernatant was precipitated by ammonium sulfate followed by dialysis against PBS. ADAM12-S containing protein solution was processed by FPLC using an anion-exchange column (GE Healthcare, Chicago, IL, USA). Collected peaks including ADAM12-S were incubated with Concanavalin A beads (GE Healthcare, Chicago, IL, USA) and eluted by α-D-mannopyracide followed by dialysis against 50 mM Tris-HCl pH7.4. GST-ephrin-A1 was purified as previously described [19]. HEK293 cells were transfected with pEB-multi-Hygro-FLAG ephrin-A1 174R. Forty-eight hours after transfection, the cells were lysed by lysis buffer (50 mM Tris–HCl pH 7.4, 150 mM NaCl, 1% Nonidet P40, and 0.5% sodium cholate) followed by an incubation with anti-DYKDDDDK tag Antibody Beads (WAKO). The beads were extensively washed with wash buffer (20 mM Tris-HCl-pH7.4, 150 mM NaCl, 0.2% Tween-20, and 0.5% Sodium Cholate). FLAG-tagged ephrin-A1 174 was eluted by DYKDDDDK peptide (0.1 mg/mL, WAKO)-containing elution buffer (20 mM Tris-HCl pH7.4, 150 mM NaCl). The eluent was dialyzed against PBS and concentrated by amicon columns (MerckMillipore, Burlington, MA, USA). The concentration was determined by a spectrophotometer (Nano drop, ThermoFisher Scientific, San Jose, CA, USA). 

### 4.4. Cell Culture

3LL cells were obtained from the Cell Resource Center for Biomedical Research, Institute of Development, Aging and Cancer, Tohoku University and cultivated in RPMI supplemented with 10% fetal bovine serum and penicillin/streptomycin. Chinese hamster ovary (CHO) and human embryonic kidney (HEK) 293T cells were purchased from ATCC and cultivated with DMEM/F-12 or DMEM (Fujifilm Wako, Osaka, Japan) supplemented with 10% fetal bovine serum and penicillin/streptomycin. LLC cells were obtained from RIKEN bioresource research center and cultivated with 10% fetal bovine serum and penicillin/streptomycin. 3LL cells were transfected with pEGFP-N3 empty vector and selected with 400 μg/mL G418 and sorted EGFP-positive population (MoFlo, Beckman Coulter Diagnostics, CA, USA). HEK293T cells were transfected with pDsRed-EphA1 and selected with 1.2 mg/mL G418. DsRed-positive cells were sorted with a cell sorter as described above.

### 4.5. Electrophoresis and in-Gel Digestion

GST-ephrin-A1 was incubated with recombinant ADAM12 followed by SDS-PAGE to separate cleaved- and uncleaved-ephrin-A1. Bands of cleaved-ephrin-A1, stained with CBB, were cut from a gel. The gel pieces were washed three times with 50 mM NH_4_HCO_3_ containing 50% methanol, incubated with 0.5 mL of 10 mM DTT in 50 mM NH_4_HCO_3_ at 60 °C for 60 min, and then with 0.5 mL of 50 mM acrylamide in 50 mM NH_4_HCO_3_ at 25 °C for 30 min. The gel pieces were further washed twice with water, twice with ACN, dried in air, and rehydrated with 30 μL of 50 mM NH_4_HCO_3_ buffer prepared with 65% ^18^O-labeled water (98 atom % ^18^O, Taiyo Nippon Sanso, Tokyo, Japan), and then, 1 μg of lysylend-peptidase (LEP) was added and incubated at 37 °C for 12 h. The resultant digested peptides were recovered in 50% ACN containing 0.1% TFA, dried in a speed vac, and dissolved in 20 μL of 20% ACN containing 0.1% TFA. An aliquot was mixed with a matrix solution (7 mg/mL of CHCA in 50% aqueous ACN containing 0.1% TFA, dried in air and subjected to MALDI-MS. 

### 4.6. Μatrix-Assisted Laser Desorption Ionization Mass Spectrometry (MALDI-MS)

MALDI-MS and MS/MS were carried out with a 4800 MALDI-TOF/TOF mass spectrometer (Applied Biosystems, Framingham, MA, USA). All mass spectra were obtained by averaging 2500 laser shots from each sample well in the positive-ion mode. For the MS/MS measurements, the metastable suppressor and CID gas were both set at “ON”. The entire process was controlled using 4000 series Explorer software (version 3.6; Applied Biosystems). Data were processed using Data Explorer software (version 4.8; Applied Biosystems). The MS/MS spectra were obtained with a fully automatic workflow using a 4800 MALDI-TOF/TOF analyzer. The resultant MS/MS spectra were processed using Mascot Distiller (Matrix Science Ltd, London, UK) and subjected to a database search by Mascot (MS/MS Ions Search).

### 4.7. Synthesis of KB-R7785 and UniPR1331

KB-R7785 and UniPR1331 were synthesized as described previously [23,28]. The purities of synthesized KB-R7785 and UniPR1331 were evaluated by HPLC and were more than 99%.

### 4.8. Hibit Luminescence Assay

HiBiT reaction was performed based on an instruction manual (HiBiT Extracellular Detection, Bio-Rad Laboratories, CA, USA), and the luminescence was measured by a luminometer (GloMax96, Bio-Rad Laboratories). 

### 4.9. Cell-Spreading Assay

An eight-well chamber slide was coated with fibronectin (5 μg/mL) with or without ephrin-A1-Fc (1 μg/mL) or ephrin-A1 174R (1 μg/mL). EphA1-expressing cells (5 × 10^4^ cells/well) were seeded onto the well and incubated for 30 min in a CO_2_ incubator. The number of cell (>100 cells) attached to the bottom of the wells was counted under a fluorescence microscope (ECLIPSE Ti2, Nikon, Tokyo, Japan) after washing out floating cells by PBS.

### 4.10. Dissociation Assay of the Epha1/Ephrin-A1 Complex

YFP-ephrin-A1-expressing HEK293 cells (1 × 10^5^ cells) were co-cultured with EphA1-DsRed-expressing HEK293 cells (1 × 10^5^ cells) in a glass bottom culture dish (35 mm, IWAKI, Shizuoka, Japan) overnight. The cells were stimulated by TGF-β (5 ng/mL), and time-lapse images were taken under a confocal laser scanning microscope (LSM710, ZEISS, Oberkochen, Germany).

### 4.11. Animal Study

C57BL/6J mice were purchased from CLEA (Tokyo, Japan). The mice were used for experiments at 8–10 weeks old. C57BL/6J mice were subcutaneously inoculated with 2 × 10^5^ 3LL-EGFP cells and intraperitoneally injected with 20 μg of KB-R7785 from three days after tumor inoculation until sacrificed each other day. KB-R7785 dissolved in DMSO was mixed with 0.5% CMC. Tumor volumes were weighed with an electronic weighing instrument. UniPR1331 was dissolved in 0.5% CMC and administrated per os six days per week. Tumor volumes were measured with a caliper and calculated as (length × width^2^)/2 [19]. 

### 4.12. Statistical Analysis

Data are expressed as means±s.d. or sem. Comparisons between two groups were performed with the two-tailed, paired Student’s *t*-test. In all experiments, *p* < 0.05 was considered statistically significant.

## Supplementary Materials

The following are available online at https://www.mdpi.com/1422-0067/22/5/2480/s1, Figure S1: The amino acid sequence of mouse ephrin-A1. Mouse ephrin-A1 purified from urine was digested with LEP. Lysine digested by LEP is shown in red. Underlined peptide was found by a mass spectrometric analysis.
