# Supplementary material for: Analysis of ADAM12-Mediated Ephrin-A1 Cleavage and Its Biological Functions
Source: Int J Mol Sci. 2021 Mar 1;22(5):2480. doi: 10.3390/ijms22052480 (PMC7957476; doi:10.3390/ijms22052480)
Supplement: Supplementary file 1 [file ijms-22-02480-s001.pdf]

Supplementary Figure 1

1	10	21	31	41
MEFLWAPLLG	LCCSLAAADR	HIVFWNSSNP	KFREEDYTVH	VQLNDYLDII
51	61	71	81	91
CPHYEDDSVA	DAAMERYTLY	MVEHQEYVAC	QPQSKDQVRW	NCNRPSAKHG
101	111	121	131	141
PEKLSEKFQR	FTPFILGKEF	KEGHSYYYIS	KPIYHQESQC	LKLKVTVNGK
151	161	171	181	191
ITHNPQAHVN	PQEKRLQADD	PEVQVLHSIG	YSAAPRLFPL	VWAVLLLPLL
201				
LLQSQ				
